# Google's Project Nightingale highlights the necessity of data science ethics review

**DOI:** 10.15252/emmm.202012053

**Published:** 2020-02-17

**Authors:** Christophe Olivier Schneble, Bernice Simone Elger, David Martin Shaw

**Affiliations:** ^1^ Institute of Biomedical Ethics University of Basel Basel Switzerland; ^2^ Faculty of Medicine University Center of Legal Medicine University of Geneva Geneva Switzerland; ^3^ Department of Health, Ethics and Society Care and Public Health Research Institute Maastricht University Maastricht The Netherlands

**Keywords:** S&S: Ethics

## Abstract

On November 14 last year, the British *Guardian* published an account from an anonymous whistleblower at Google, accusing the company of misconduct in regard to handling sensitive health data. The whistleblower works for Project Nightingale, an attempt by Google to get into the lucrative US healthcare market, by storing and processing the personal medical data of up to 50 million customers of Ascension, one of America's largest healthcare providers. As the *Wall Street Journal* had already reported 3 days earlier, and as the whistleblower confirmed, neither was the data anonymized when transmitted from Ascension nor were patients or their doctors notified, let alone asked for consent to sharing their data with Google (Copeland, 2019; Pilkington, 2019). As a result, Google employees had full access to non‐anonymous patient health data. Google Health chief David Feinberg commented that all Google employees involved had gone through medical ethics training and were approved by Ascension (Feinberg, 2019).

## Graphical abstract

The Google's Project Nightingale is meant to collect and analyse the detailed personal‐health information of millions of people across the USA. As discussed by C.O. Schneble, B.S. Elger and D.M. Shaw, without thorough ethical measures, such undertaking is at risk of misconduct.

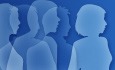

Although Nightingale violated no laws regarding health data, the case raised fears over privacy. Ethically speaking, Ascension and Google ignored various standards for handling personal and sensitive data (Zook *et al*, 2017). First, this was clearly a breach of confidentiality as patients trusted the hospitals that their health data would be managed with the greatest respect for privacy. Second, patients were not asked for consent to share their data with Google for storage and processing. Third, patients’ privacy was seriously disrespected, because data were not anonymized prior to its transfer to Google.

## Complex legal regulations

Many privacy/data protection laws regard health‐related data as special category that requires a higher level of protection than conventional data. In the USA, the Health Insurance Portability and Accountability Act (HIPAA) offers some legal protection. However, the law contains a loophole as it allows hospitals or healthcare providers to disclose information to business associates for further processing or quality improvement. In the EU, the General Data Protection Regulation (GDPR) defines health data as a special category of data, the processing of which is prohibited outside the EU unless explicit consent has been given (Art. 9 lit. 1). Overall, the legal situation regarding the protection of health data remains complex and further clarification through court cases will take time.

In the meantime, ethics continues to play an important role to protect the rights of subjects whose data are being collected, processed, shared and used. Some basic principles provide a stopgap measure until the law catches up with broader conceptions of health data. Indeed, years ago the collection and storage of personal health data were very limited, compared to today's omnipresent data gathering by institutional and commercial entities, social media and smartphone apps. The concept of health data as a distinct category is also being challenged, as various research projects have used “social media data” to derive and predict health‐relevant issues such as risk of depression (Reece & Danforth, 2017). This raises the question of whether all personal data should be regarded as health data (Schneble *et al*, 2019). In any case, the broadening notion of health data makes it all the more important that researchers respect ethical principles and that appropriate oversight mechanisms for data science are in place.

## Towards responsible data science

Scientists need to keep several points in mind when using personal health data for research. Below is some minimal guidance that can be used to evaluate such research projects and to stay clear of potential legal challenges.

### Transparency

It must be clear for patients and participants for which purposes their data are being used and where and by whom they are used and processed. Thus, health data derived in the clinic need to remain in the context of treatment unless the patient has agreed to share it for further research.

### Explicit consent

The storing and processing of health data are always subject to prior explicit consent by the data subject (patient). Explicit consent means that patients need to be informed for which purposes their data are being used, where it is being stored and how their data will be used in the future before the project starts. Patients also have the right to decide whether data are shared in anonymized or identifiable form. Last but not least, patients should be able to have their data corrected or to withdraw from a study.

### Data anonymization

Traditional anonymization techniques are increasingly being challenged by novel technologies such as machine learning, so scientists need to pay more attention to this topic. Simply cutting out birth date, zip code and sex have been proven to be ineffective. Using more up‐to‐date and complex methods such as k‐Anonymity is a better solution, albeit still not an absolutely certain method for ensuring anonymity.

### Ethical reflection

Researchers should ask themselves broader ethical questions about their research. These questions are not new and have been the focus of ethically sound research for decades: Have patients agreed to their data being shared? Does the patient benefit from his or her data being shared? A negative answer does not prevent data sharing and processing per se, but direct patient benefits make it easier to justify the research. Who else would benefit from data sharing and do these benefits justify the risks? Is it necessary to share identifiable data or can research be carried out with pseudonymized/anonymized data? Has enough effort been put into maintaining privacy, including a strict anonymization regime? Is trust/confidentiality between health institution and the patients maintained?

The Association of Internet Research has recently published new ethical guidelines that offer further challenging questions to consider (Franzke *et al*, 2019).

## The urgent need for IRB review

While researchers should do more to consider ethical issues themselves, Project Nightingale accentuates the urgent need for institutional review boards and research ethics committees (IRBs and RECs) to evaluate data‐driven research. While discussion continues and the ethical principles that should govern best practices in data science and AI research are being developed, such research should not be exempted from IRB evaluation. In the past, such bodies have always been challenged by novel technologies, but have proven their effectiveness at achieving a balance between the interests of science and those who participate directly in research or whose data and samples are used for research. Furthermore, depending on the jurisdiction and the topic, health research has to undergo mandatory IRB evaluation in both academic and commercial settings. However, in many cases data science lies outside the scope of it. Universities have therefore set up IRB structures to evaluate this type of research, and many journals also request to see the approval by an IRB or REC.

In cases where research at the corporate level is not subject to IRB review—either because the jurisdiction does not require it or because such bodies do not exist—it might lead to a fundamental inequality between public and private research entities. It therefore remains dubious that many large “data companies” have not introduced such review bodies in their organization. Implementing ethical review is important to ensure transparency and a long‐term trusted partnership between companies and the “customers” that we all are.
